# Some Order Preserving Inequalities for Cross Entropy and Kullback–Leibler Divergence

**DOI:** 10.3390/e20120959

**Published:** 2018-12-12

**Authors:** Mateu Sbert, Min Chen, Jordi Poch, Anton Bardera

**Affiliations:** 1 College of Intelligence and Computing, Tianjin University, Tianjin 300350, China; 2 Graphics and Imaging Laboratory, University of Girona, Campus Montilivi, 17003 Girona, Spain; 3 Department of Engineering Science, University of Oxford, Oxford OX1 3PJ, UK

**Keywords:** cross entropy, Kullback–Leibler divergence, likelihood, Kolmogorov mean, generalized mean, weighted mean, stochastic dominance, stochastic order

## Abstract

Cross entropy and Kullback–Leibler (K-L) divergence are fundamental quantities of information theory, and they are widely used in many fields. Since cross entropy is the negated logarithm of likelihood, minimizing cross entropy is equivalent to maximizing likelihood, and thus, cross entropy is applied for optimization in machine learning. K-L divergence also stands independently as a commonly used metric for measuring the difference between two distributions. In this paper, we introduce new inequalities regarding cross entropy and K-L divergence by using the fact that cross entropy is the negated logarithm of the weighted geometric mean. We first apply the well-known rearrangement inequality, followed by a recent theorem on weighted Kolmogorov means, and, finally, we introduce a new theorem that directly applies to inequalities between K-L divergences. To illustrate our results, we show numerical examples of distributions.

## 1. Introduction

Cross entropy and Kullback–Leibler (K-L) divergence are applied to and discussed in studies on machine learning [1], visualization (e.g., to interpret the pipeline of visualization using the cost–benefit model [2] and several other applications [3]), computer graphics [4], and many other fields. Cross entropy and K-L divergence are two fundamental quantities of information theory. Cross entropy gives the average code length needed to represent one distribution by another distribution, and the excess code needed over the optimal coding is given by the K-L divergence. As cross entropy is just the negated logarithm of likelihood, minimizing cross entropy means maximizing likelihood, and thus, cross entropy is widely used now for optimization in machine learning. K-L divergence also stands independently as a widely used metric for measuring the difference between two distributions, and it appears in many theoretical results. As examples, the definition of mutual information between two variables is a K-L divergence, and the absolute difference of K-L divergences with respect to a third distribution was recently used to define a family of metrics [5].

Recently, order invariance for inequalities between means was presented [6]. Since likelihood is the weighted geometric mean of the representing distribution weighted by the data distribution, likelihood and thus cross entropy will share the order invariance properties too. Order invariance properties are characterized by the stochastic order between weights or data distributions [7]. Here, we further develop this idea in order to compare representations of different data by the same distribution, and we extend it to a new order, K-L dominance, that allows us to define inequalities between representations of the same data by different distributions. Finally, we establish the connection between the two orders. We also give numerical examples to illustrate the theoretical results.

This paper is organized as follows. After this introduction, in Section 2, we recapitulate the meaning of cross entropy and its relationship to likelihood and K-L divergence, and we obtain the first theoretical results based on the rearrangement inequality. Next, in Section 3, we apply the invariance results for weighted means to cross entropy. In Section 4, we introduce a new order, K-L dominance, that allows us to establish inequalities between K-L divergences and give the relationship between stochastic and K-L order. Finally, in Section 5, we present our conclusions and future work.

## 2. Cross Entropy as a Measure of Goodness of Representation

Consider two probability distributions, ${\left\{\alpha_{k}\right\}}_{k=1}^{n}$, ${\left\{x_{k}\right\}}_{k=1}^{n}$, for all *k* $\alpha_{k}\geq 0$, $x_{k}>0$, $\sum_{k}\alpha_{k}=\sum_{k}x_{k}=1$ (when not explicit, the sum limits will be understood to be between 1 and *n*). Cross entropy $CE(\alpha_{k},x_{k})$ (to avoid cluttered notation we will write $CE(\alpha_{k},x_{k})$ instead of $CE(\left\{\alpha_{k}\right\},\left\{x_{k}\right\})$) is defined as $CE\left(\alpha_{k},x_{k}\right)=\sum_{k}\alpha_{k}\log\frac{1}{x_{k}}=-\sum_{k}\alpha_{k}\log x_{k}$ and can be written as $CE\left(\alpha_{k},x_{k}\right)=H\left(\alpha_{k}\right)+D_{\mathrm{KL}}\left(\alpha_{k},x_{k}\right)$ (similar notation to $CE(\alpha_{k},x_{k})$), where $H\left(\alpha_{k}\right)=-\sum_{k}\alpha_{k}\log\alpha_{k}$ is the entropy of distribution $\left\{\alpha_{k}\right\}$, and $D_{\mathrm{KL}}\left(\alpha_{k},x_{k}\right)=\sum_{k}\alpha_{k}\log\frac{\alpha_{k}}{x_{k}}\geq 0$ is the Kullback–Leibler divergence of $\left\{\alpha_{k}\right\}$ and $\left\{x_{k}\right\}$ distributions. It represents the average length of code per symbol needed to represent $\left\{\alpha_{k}\right\}$ using $\left\{x_{k}\right\}$. In the context of coding, we do not let any component of $\left\{x_{k}\right\}$ be zero, as the corresponding component of $\left\{\alpha_{k}\right\}$ would not be coded; thus, $D_{\mathrm{KL}}\left(\alpha_{k},x_{k}\right)$ is well defined. The minimum code length to code $\left\{\alpha_{k}\right\}$ is obtained by taking $\left\{\alpha_{k}\right\}\equiv \left\{x_{k}\right\}$, because $D_{\mathrm{KL}}\left(\alpha_{k},\alpha_{k}\right)=0$. Minimum code length will be between $H(\alpha_{k})$ and $H(\alpha_{k})$ + 1 bit (Huffman coding, [8]).

### Cross Entropy and Likelihood

Observe that cross entropy is the negated logarithm of likelihood. Let us suppose a distribution with *n* states, which are guessed to be $\left\{x_{1},x_{2},\ldots ,x_{n}\right\}$, and a relative frequency of realizations $\left\{\alpha_{1},\alpha_{2},\ldots ,\alpha_{n}\right\}$ of the different states. That is, we guess that the distribution $\left\{x_{k}\right\}$ represents the collected data $\left\{\alpha_{k}\right\}$. Likelihood is then used as a measure of how well the distribution represents the data. The likelihood $L(\alpha_{k},x_{k})$ of $\left\{x_{k}\right\}$ is the true distribution of the data with relative frequency $\left\{\alpha_{k}\right\}$, and $L=x_{1}^{\alpha_{1}}x_{2}^{\alpha_{2}}\ldots x_{n}^{\alpha_{n}}=\Pi_{k}x_{k}^{\alpha_{k}}$. Observe that $L(\alpha_{k},x_{k})$ is the weighted geometric mean of $\left\{x_{1},x_{2},\ldots ,x_{n}\right\}$ with weights $\left\{\alpha_{1},\alpha_{2},\ldots ,\alpha_{n}\right\}$. The likelihood represents the probability of $\left\{x_{k}\right\}$ being the true distribution of the known data $\left\{\alpha_{k}\right\}$. As $CE\left(\alpha_{k},x_{k}\right)=-\log L\left(\alpha_{k},x_{k}\right)$, maximizing the likelihood is equivalent to minimizing the cross entropy.

Now, let us suppose we take an arbitrary $\left\{x_{k}\right\}$ distribution to code $\left\{\alpha_{k}\right\}$. Without loss of generality, we will consider two or more sequences to be equally ordered, or in the same order, when, by the same permutation of indexes, we can make it such that all the sequences are increasing. We can state the following theorem.

**Theorem** **1.** *Consider the distribution $\left\{\alpha_{k}\right\}$ and the sequence of strictly positive numbers $\left\{x_{1},x_{2},\ldots ,x_{n}\right\}$, $\sum_{k}x_{k}=1$. When $\left\{x_{1},x_{2},\ldots ,x_{n}\right\}$ is permuted so that $\left\{x_{k}\right\}$ is ordered in the same order as $\left\{\alpha_{k}\right\}$, then the cross entropy $CE(\alpha_{k},x_{k})$ takes a global minimum, and, correspondingly, the likelihood $L(\alpha_{k},x_{k})$ takes a global maximum.*

**Proof.**  Using the rearrangement inequality [6,9] and the definition of cross entropy, the minimum of the sum of the product $\Pi_{k}\alpha_{k}\log\frac{1}{x_{k}}$ is realized when the two sequences are ordered inversely to each other. That means the $\left\{\log x_{k}\right\}$ sequence will be ordered as the $\left\{\alpha_{k}\right\}$ sequence, and, by the monotonicity of the logarithmic function, the $\left\{x_{k}\right\}$ sequence will be ordered as the $\left\{\alpha_{k}\right\}$ sequence. □

As $\left\{\alpha_{k}\right\}$, and thus $H(\alpha_{k})$, is fixed, $D_{\mathrm{KL}}\left(\alpha_{k},x_{k}\right)$ also takes the global minimum when $\left\{x_{k}\right\}$ is ordered as $\left\{\alpha_{k}\right\}$. Also, when there are elements repeated in $\left\{\alpha_{k}\right\}$, there can be non-ordered $\left\{x_{k}\right\}$ distributions with the same minimum cross entropy value. The extreme case is when $\alpha_{k}=1/n$ for all *k*, and all orderings of $\left\{x_{k}\right\}$ will give the same result.

Observe that by Theorem 1, if we have a series of distributions ${\left\{\alpha_{t,k}\right\}}_{t=1}^{m}$ that are all ordered in the same order, which we can consider to be increasing, and want to represent them with a single distribution $\left\{x_{k}\right\}$—to be chosen by permuting the elements in $\left\{x_{1},x_{2},\ldots ,x_{n}\right\}$—then the best distribution will be the one for which we select the elements in $\left\{x_{1},x_{2},\ldots ,x_{n}\right\}$ in increasing order too.

**Example** **1.** *For any increasing sequence $\left\{\alpha_{k}\right\}$, we have that*

*1.*  $CE\left(\left\{\alpha_{k}\right\},\left\{2/9,3/9,4/9\right\}\right)\leq CE\left(\left\{\alpha_{k}\right\},\left\{4/9,3/9,2/9\right\}\right),$
*2.*  *$CE\left(\left\{\alpha_{k}\right\},\left\{2/9,3/9,4/9\right\}\right)\leq CE\left(\left\{\alpha_{k}\right\},\left\{4/9,2/9,3/9\right\}\right),$ and so on.*

However, what happens when we have to choose between two distributions, $\left\{x_{k}\right\}$ and $\left\{x_{k}^{\prime }\right\}$, to represent a set of increasing distributions, ${\left\{\alpha_{t,k}\right\}}_{t=1}^{m}$? Can we find which one is better? Section 4 provides some answers to this. Before answering this question, we introduce the concept of stochastic dominance in Section 3.

## 3. First Stochastic Order Dominance

When for all increasing distributions $\left\{x_{k}\right\}:\sum_{k}\alpha_{k}^{\prime }x_{k}\leq \sum_{k}\alpha_{k}x_{k}$, we say that $\left\{\alpha_{k}\right\}$ stochastically dominates $\left\{\alpha_{k}^{\prime }\right\}$, and we denote this relation as $\left\{\alpha_{k}\right\}{≻}_{st}\left\{\alpha_{k}^{\prime }\right\}$ [7]. This is a partial order on the set of distributions.

The necessary and sufficient conditions for a sequence $\left\{\alpha_{k}\right\}$ to stochastically dominate another one $\left\{\alpha_{k}^{\prime }\right\}$ are given in the following theorem [6].

**Theorem** **2.** *Given that the sequences $\left\{\alpha_{k}\right\}$ and $\left\{\alpha_{k}^{\prime }\right\}$ are positive and add up to 1, the following conditions are equivalent:*

*(1)*  *The sequence $\left\{\alpha_{k}\right\}$ stochastically dominates the sequence $\left\{\alpha_{k}^{\prime }\right\}$*
*(2)*  (1) $$\begin{array}{ccc} \alpha_{1}^{\prime } & \geq & \alpha_{1}\\ \alpha_{1}^{\prime }+\alpha_{2}^{\prime } & \geq & \alpha_{1}+\alpha_{2}\\ \ldots & \geq & \ldots \\ \alpha_{1}^{\prime }+\alpha_{2}^{\prime }+\ldots +\alpha_{n}^{\prime } & = & \alpha_{1}+\alpha_{2}+\ldots +\alpha_{n} \end{array}$$
*(3)*  (2) $$\begin{array}{ccc} \alpha_{n}^{\prime } & \leq & \alpha_{n}\\ \alpha_{n}^{\prime }+\alpha_{n-1}^{\prime } & \leq & \alpha_{n}+\alpha_{n-1}\\ \ldots & \leq & \ldots \\ \alpha_{n}^{\prime }+\alpha_{n-1}^{\prime }+\ldots +\alpha_{1}^{\prime } & = & \alpha_{n}+\alpha_{n-1}+\ldots +\alpha_{1} \end{array}$$
*(4)*  *For all increasing sequences $\left\{x_{k}\right\}$ and for any f(x) strictly monotonous function, $f^{-1}\left(\sum_{k}\alpha_{k}^{\prime }f\left(x_{k}\right)\right)\leq f^{-1}\left(\sum_{k}\alpha_{k}f\left(x_{k}\right)\right)$ (quasi-arithmetic, or Kolmogorov, mean), whenever f(x) is applicable.*
*(5)*  *There exists a strictly monotonous function f(x) such that for all increasing sequences $\left\{x_{k}\right\}$:*
  (3) $$f^{-1}\left(\sum_{k}\alpha_{k}^{\prime }f\left(x_{k}\right)\right)\leq f^{-1}\left(\sum_{k}\alpha_{k}f\left(x_{k}\right)\right)$$
  *(quasi-arithmetic, or Kolmogorov, mean), whenever f(x) is applicable.*

A sufficient condition for $\left\{\alpha_{k}\right\}{≻}_{st}\left\{\alpha_{k}^{\prime }\right\}$ is given by the following theorem [6,10].

**Theorem** **3.** *Given the distributions $\left\{\alpha_{k}\right\},\left\{\alpha_{k}^{\prime }\right\}$, a sufficient condition for $\left\{\alpha_{k}\right\}{≻}_{st}\left\{\alpha_{k}^{\prime }\right\}$ is that whenever i≤j, then $\frac{\alpha_{i}^{\prime }}{\alpha_{j}^{\prime }}\geq \frac{\alpha_{i}}{\alpha_{j}}$.*

By Theorem 3, if $\left\{\alpha_{k}\right\}$ is increasing, then $\left\{\alpha_{k}\right\}{≻}_{st}\left\{1/n\right\}$, where {1/n} is the uniform distribution; if $\left\{\alpha_{k}\right\}$ is decreasing, then $\left\{1/n\right\}{≻}_{st}\left\{\alpha_{k}\right\}$; if $\left\{\alpha_{k}\right\}$ is increasing and $\left\{\alpha_{k}^{\prime }\right\}$ is decreasing, then $\left\{\alpha_{k}\right\}{≻}_{st}\left\{\alpha_{k}^{\prime }\right\}$.

Observe now that, by condition (5) of Theorem 2, if Equation (3) applies for one strictly monotonous function, then, for the equivalence between conditions (4) and (5), it applies for any other strictly monotonous function. Then, by the definition of cross-entropy,

**Theorem** **4.** *Given two distributions $\left\{\alpha_{k}\right\}$ and $\left\{\alpha_{k}^{\prime }\right\}$, $\left\{\alpha_{k}\right\}{≻}_{st}\left\{\alpha_{k}^{\prime }\right\}$ if and only if for all increasing distributions $\left\{x_{k}\right\}$, $CE\left(\alpha_{k},x_{k}\right)\leq CE\left(\alpha_{k}^{\prime },x_{k}\right)$ (or $L\left(\alpha_{k},x_{k}\right)\geq L\left(\alpha_{k}^{\prime },x_{k}\right)$).*

Theorem 4 means that, if $\left\{\alpha_{k}\right\}{≻}_{st}\left\{\alpha_{k}^{\prime }\right\}$, then, taking all possible increasing representations $\left\{x_{k}\right\}$, the code length for $\left\{\alpha_{k}\right\}$ will be shorter than for $\left\{\alpha_{k}^{\prime }\right\}$, and the likelihood for $\left\{\alpha_{k}\right\}$ will be bigger than the likelihood for $\left\{\alpha_{k}^{\prime }\right\}$. Observe also that if $\left\{\alpha_{k}\right\}$ is an increasing sequence, then $\left\{\alpha_{k}\right\}{≻}_{st}\left\{1/n\right\}$; thus, the representation of $\left\{\alpha_{k}\right\}$ by an increasing distribution $\left\{x_{k}\right\}$ will be shorter than the representation of uniform distribution {1/n}, and the likelihood of $\left\{\alpha_{k}\right\}$ will be bigger than that of {1/n}.

**Corollary** **1.** *For any increasing distributions $\left\{x_{k}\right\}$ and $\left\{\alpha_{k}\right\}$, we have that $CE\left(\alpha_{k},x_{k}\right)\leq CE\left(1/n,x_{k}\right)$ (or $L\left(\alpha_{k},x_{k}\right)\geq L\left(1/n,x_{k}\right))$.*

Indeed, the above result is valid too if, by an index permutation, we can bring $\left\{x_{k}\right\}$, $\left\{\alpha_{k}\right\}$ to be in the same order. Thus,

**Corollary** **2.** *For any distributions $\left\{x_{k}\right\}$ and $\left\{\alpha_{k}\right\}$ that are ordered equally, we have that $CE\left(\alpha_{k},x_{k}\right)\leq CE\left(1/n,x_{k}\right)$.*

Now, suppose $\left\{\alpha_{k}\right\}$ is decreasing; then, $\left\{1/n\right\}{≻}_{st}\left\{\alpha_{k}\right\}$. Also, for any distribution $\left\{x_{k}\right\}$, $CE\left(1/n,x_{k}\right)\geq CE\left(1/n,1/n\right)=\log n$, and thus, the following corollary,

**Corollary** **3.** *For any increasing distribution $\left\{x_{k}\right\}$ and decreasing distribution $\left\{\alpha_{k}\right\}$, we have that $\log n\leq CE\left(1/n,x_{k}\right)\leq CE\left(\alpha_{k},x_{k}\right)$.*

Also, by reordering the indexes, Corollary 3 can be extended to

**Corollary** **4.** *For any distributions $\left\{x_{k}\right\}$ and $\left\{\alpha_{k}\right\}$ that are inversely ordered to each other, we have that $\log n\leq CE\left(1/n,x_{k}\right)\leq CE\left(\alpha_{k},x_{k}\right)$.*

Observe now that if $\left\{\alpha_{k}\right\}$ is increasing and $\left\{\alpha_{k}^{\prime }\right\}$ is decreasing, then, $\left\{\alpha_{k}\right\}{≻}_{st}\left\{\alpha_{k}^{\prime }\right\}$, and thus,

**Corollary** **5.** *For any increasing distribution $\left\{\alpha_{k}\right\}$ and decreasing distribution $\left\{\alpha_{k}^{\prime }\right\}$, we have that for any increasing distribution $\left\{x_{k}\right\}$, $CE\left(\alpha_{k},x_{k}\right)\leq CE\left(\alpha_{k}^{\prime },x_{k}\right)$.*

Corollary 5 is also a direct consequence of Corollaries 1 and 3.

**Example** **2.** *We have:*

*1.*  $\left\{1/4,1/4,1/2\right\}{≻}_{st}\left\{1/3,1/3,1/3\right\}$
*2.*  {1/2,1/4,1/4}≻st{1/3,1/3,1/3}
*3.*  $\left\{1/3,1/3,1/3\right\}{≻}_{st}\left\{1/2,1/4,1/4\right\}$
*4.*  {1/2,1/4,1/4}≻st{1/5,3/5,1/5}
*5.*  {1/5,3/5,1/5}≻st{1/2,1/4,1/4}

**Figure 1:**
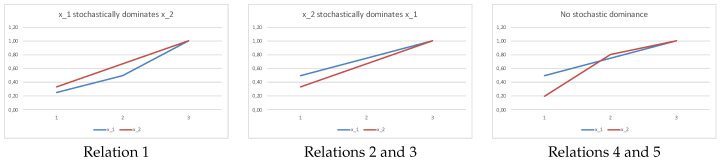
Plotting the sums in Equation (1) for relations 1–5 in Example 2.

In Figure 1, we plot the sums in Equation (1) corresponding to relation 1 and relations 2–3, 4–5 in Example 2. Observe that the sums in Equation (1) correspond to the cumulative distribution function (cdf). A first-order stochastic dominance implies that there is no crossing of the respective cdf’s.

### Discussion

We show above how the interpretation of cross entropy as a weighted mean allows for obtaining interesting order invariance results for the cross entropy of two data distributions represented by the same distribution (correspondingly for likelihood). Since, in our results, the distribution of the data is variable while the representative distribution is fixed, the resulting cross entropy depends on both entropy and K-L divergence. In the next section, we consider the data fixed and the representative distribution variable; thus, the comparison of cross entropies of a given data distribution for two representative distributions will be equivalent to the comparison of their K-L divergences.

## 4. A New Partial Order: K-L Dominance

We extend here the results of Section 2. We first define a new partial order between distributions and call it K-L dominance.

**Definition** **1.** *K-L dominance. We say that distribution $\left\{x_{k}\right\}$ K-L-dominates distribution $\left\{x_{k}^{\prime }\right\}$, written symbolically as $\left\{x_{k}\right\}{≻}_{\mathrm{KL}}\left\{x_{k}^{\prime }\right\}$, when, for all increasing distributions $\left\{\alpha_{k}\right\}$, $D_{\mathrm{KL}}\left(\alpha_{k},x_{k}\right)\leq D_{\mathrm{KL}}\left(\alpha_{k},x_{k}^{\prime }\right)$.*

Observe that {xk}≻KL{xk′}⇔{xk}≻st{xk′}, because, in general, DKL(αk,xk)≤DKL(αk,xk′)⇔DKL(xk,αk)≤DKL(xk′,αk).

From the definition of cross entropy, $\left\{x_{k}\right\}{≻}_{\mathrm{KL}}\left\{x_{k}^{\prime }\right\}$ is equivalent to—for all increasing $\left\{\alpha_{k}\right\}$—$CE\left(\alpha_{k},x_{k}\right)\leq CE\left(\alpha_{k},x_{k}^{\prime }\right)$, and thus, for all increasing $\left\{\alpha_{k}\right\}$ sequences, coding them with $\left\{x_{k}\right\}$ will always generate a shorter codification than coding them with $\left\{x_{k}^{\prime }\right\}$.

The following theorem holds:

**Theorem** **5.** *A necessary and sufficient condition for distributions $\left\{x_{k}\right\}$, $\left\{x_{k}^{\prime }\right\}$ to hold $\left\{x_{k}\right\}{≻}_{\mathrm{KL}}\left\{x_{k}^{\prime }\right\}$ (equivalent to—for all increasing distributions $\left\{\alpha_{k}\right\}$—$D_{\mathrm{KL}}\left(\alpha_{k},x_{k}\right)\leq D_{\mathrm{KL}}\left(\alpha_{k},x_{k}^{\prime }\right)$, or $CE\left(\alpha_{k},x_{k}\right)\leq CE\left(\alpha_{k},x_{k}^{\prime }\right)$) or $L\left(\alpha_{k},x_{k}\right)\geq L\left(\alpha_{k},x_{k}^{\prime }\right)$ is the following:*

(4) $$\begin{array}{ccc} x_{n}^{\prime } & \leq & x_{n}\\ x_{n}^{\prime }x_{n-1}^{\prime } & \leq & x_{n}x_{n-1}\\ \cdots & \leq & \cdots \\ x_{n}^{\prime }x_{n-1}^{\prime }\ldots x_{1}^{\prime } & \leq & x_{n}x_{n-1}\ldots x_{1} \end{array}$$

Observe that Theorem 1 is a particular case of Theorem 5, as a sequence rearranged in increasing order will always fill the second members of inequalities in Equation (4) with respect to all other rearrangements. Thus, an increasing distribution $\left\{x_{k}\right\}$ K-L-dominates all the distributions obtained by permuting the values of $\left\{x_{k}\right\}$. Observe also that Equation (4) condition makes the ${≻}_{\mathrm{KL}}$ order reflexive, antisymmetric, and transitive, and thus, it is a partial order. To prove Theorem 5, let us consider first the following lemma:

**Lemma** **1.** *Consider the sequences of n numbers $\left\{w_{k}\right\}$ and $\left\{y_{k}\right\}$. Then, conditions (1) and (2) are equivalent:*

*(1)*  *for any sequence of n positive numbers in increasing order $\left\{z_{k}\right\}$, the following inequality holds:*
  (5) $$\sum_{k=1}^{n}w_{k}z_{k}\leq \sum_{k=1}^{n}y_{k}z_{k},$$
*(2)*  *(2) the following inequalities hold:*
  (6) $$\begin{array}{ccc} w_{n} & \leq & y_{n}\\ w_{n}+w_{n-1} & \leq & y_{n}+y_{n-1}\\ \cdots & \leq & \cdots \\ w_{n}+\ldots +w_{2} & \leq & y_{n}+\ldots +y_{2} \end{array}$$
  (7) $$\begin{array}{ccc} w_{1}+\ldots +w_{n-1}+w_{n} & \leq & y_{1}+\ldots +y_{n-1}+y_{n}. \end{array}$$

**Proof.**  That (1)⇒(2) is immediate, considering, respectively, the $z_{k}$ sequences {0,0,…,1},{0,0,…,1,1},…,{0,1,…,1},{1,1,…,1}. Let us see now that (2)⇒(1): Define for 1≤n, $A_{k}=\sum_{j=1}^{k}y_{n-j+1},A_{k}^{\prime }=\sum_{j=1}^{k}w_{n-j+1}$, and $A_{0}=A_{0}^{\prime }=0$, $z_{0}=0$. Observe that condition (2) is equivalent to—for all *k*—$A_{k}-A_{k}^{\prime }\geq 0$. Observe also that the $\left\{z_{k}\right\}$ sequence augmented with $z_{0}$ is still increasing. We have

(8) $$\begin{array}{ccc} & & \sum_{k=1}^{n}y_{n-k+1}z_{n-k+1}-\sum_{k=1}^{n}w_{n-k+1}z_{n-k+1}\\ & = & \sum_{k=1}^{n}\left(y_{n-k+1}-w_{n-k+1}\right)z_{n-k+1}\\ & = & \sum_{k=1}^{n}\left(A_{k}-A_{k-1}-{A^{\prime }}_{k}+{A^{\prime }}_{k-1}\right)z_{n-k+1}\\ & = & \sum_{k=1}^{n}\left(A_{k}-{A^{\prime }}_{k}\right)z_{n-k+1}-\sum_{k=1}^{n}\left(A_{k-1}-{A^{\prime }}_{k-1}\right)z_{n-k+1}\\ & = & \sum_{k=1}^{n}\left(A_{k}-{A^{\prime }}_{k}\right)z_{n-k+1}-\sum_{k=0}^{n-1}\left(A_{k}-{A^{\prime }}_{k}\right)z_{n-k}\\ & = & \sum_{k=1}^{n}\left(A_{k}-{A^{\prime }}_{k}\right)z_{n-k+1}-\sum_{k=1}^{n}\left(A_{k}-{A^{\prime }}_{k}\right)z_{n-k}\\ & = & \sum_{k=1}^{n}\left(A_{k}-{A^{\prime }}_{k}\right)\left(z_{n-k+1}-z_{n-k}\right)\geq 0, \end{array}$$

as, by hypothesis for all *k*, $A_{k}-A_{k}^{\prime }\geq 0$, and $\left\{z_{k}\right\}$ is an increasing sequence. □

The proof of Theorem 5 follows immediately from applying Lemma 1 to the inequality $\sum_{k}\alpha_{k}\log x_{k}^{\prime }\leq \sum_{k}\alpha_{k}\log x_{k}$.

The following corollary follows from observing that, by taking $\left\{\alpha_{k}\right\}\equiv \left\{x_{k}\right\}$, then, $D_{\mathrm{KL}}\left(x_{k},x_{k}\right)=0<D_{\mathrm{KL}}\left(x_{k},x_{k}^{\prime }\right)$, {xk′}≡{xk}.

**Corollary** **6.** *An increasing distribution $\left\{x_{k}\right\}$ cannot be K-L dominated by any other distribution (except trivially by itself). In particular, the uniform distribution $\left\{x_{k}\right\}\equiv \left\{1/n\right\}$ does not dominate any other increasing distribution.*

Observe now that the product $1/n^{n}$ is always bigger than or equal $\Pi_{k}x_{k}$, as the maximum value for $\Pi_{k}x_{k}$ subjected to the condition $\sum_{k}x_{k}=1$ is, for all *k*, $x_{k}=1/n$. Thus, from the last inequality in Equation (4), we have the following corollary,

**Corollary** **7.** *For all distributions $\left\{x_{k}\right\}$, {xk}≡{1/n}, we have that {xk}≻KL{1/n}.*

Observe that Corollary 7 is also a particular case of Corollary 6, considering {1/n} as an increasing distribution.

**Corollary** **8.** *The uniform distribution {1/n} K-L-dominates all decreasing distributions.*

**Proof.**  For all $\left\{\alpha_{k}\right\}$ increasing and $\left\{x_{k}\right\}$ decreasing, we apply Corollary 4, and we have $CE\left(\alpha_{k},1/n\right)=\log n\leq CE\left(\alpha_{k},x_{k}\right)$. ☐

**Example** **3.** *Some examples are:*

*1.*  $\left\{1/3,1/3,1/3\right\}{≻}_{\mathrm{KL}}\left\{4/9,3/9,2/9\right\}$
*2.*  $\left\{1/3,1/3,1/3\right\}{≻}_{\mathrm{KL}}\left\{7/9,1/9,1/9\right\}$
*3.*  {1/3,1/3,1/3}≻KL{1/9,1/9,7/9}
*4.*  $\left\{1/3,1/3,1/3\right\}{≻}_{\mathrm{KL}}\left\{4/9,2/9,3/9\right\}$
*5.*  {1/3,1/3,1/3}≻KL{3/9,2/9,4/9}
*6.*  $\left\{1/4,1/4,1/2\right\}{≻}_{\mathrm{KL}}\left\{1/4,1/2,1/4\right\}$
*7.*  $\left\{1/2,1/4,1/4\right\}{≻}_{\mathrm{KL}}\left\{5/8,2/8,1/8\right\}$

**Figure 2:**
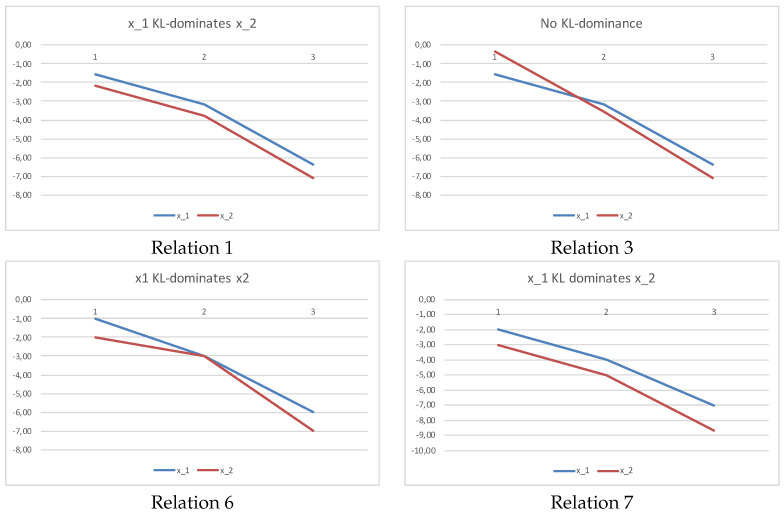
Plotting the logarithm of products in Equation (4) for relations 1, 3, 6, and 7 in Example 3.

The first and second relation are examples of Corollary 8. The third is an example of Corollary 6. The sixth is an example of Theorem 1. The fourth and fifth relations mean that the uniform distribution can both K-L dominate and non-dominate non-monotonous sequences, and we have to look for each case at the Condition (4) of Theorem 5. The seventh relation tells us that a decreasing sequence can dominate another decreasing sequence. In Figure 2, we plot the logarithm of products in Equation (4) for relations 1, 3, 6, and 7, respectively. If a sequence K-L-dominates another one, the plots do not cross, and its plot appears over that of the second sequence. Inversely, no dominance means that the plots will cross.

### 4.1. Discussion

Remember that the equivalence between cross entropy and likelihood means that the likelihood that $\left\{x_{k}\right\}$ is the true distribution, given the data distribution $\left\{\alpha_{k}\right\}$, and the average length of code using $\left\{x_{k}\right\}$ to represent $\left\{\alpha_{k}\right\}$ are related by the second quantity being the negated logarithm of the first. If you have one distribution $\left\{\alpha_{k}\right\}$ and have the choice to use $\left\{x_{1,k}\right\}$ or $\left\{x_{2,k}\right\}$ to represent it, you want to choose the one with the minimum length of code, or, alternatively, the one with maximum likelihood. In both cases, the best is the one that gives minimum Kullback–Leibler divergence. Suppose now you want to represent data $\left\{\alpha_{k}\right\}$ and have two alternative distributions to represent it, either $\left\{x_{1,k}\right\}\equiv \left\{1/n\right\}$, the uniform distribution, or $\left\{x_{2,k}\right\}$, which is ordered in the same order as $\left\{\alpha_{k}\right\}$. To fix ideas and without loss of generality, let us consider that both $\left\{\alpha_{k}\right\}$ and $\left\{x_{2,k}\right\}$ are in increasing order. It would seem reasonable to represent the data $\left\{\alpha_{k}\right\}$, which is increasing, as distribution $\left\{x_{2,k}\right\}$, which is increasing too; thus, the order is preserved. However, that would work as long as $D_{\mathrm{KL}}\left(\alpha_{k},x_{2,k}\right)<D_{\mathrm{KL}}\left(\alpha_{k},1/n\right)$; otherwise, we should choose $\left\{x_{1,k}\right\}$, the uniform distribution. This is clear from corollary 7, which tells us that, for any sequence, particularly $\left\{x_{2,k}\right\}$, there will always be increasing $\left\{\alpha_{k}\right\}$ sequences such that $D_{\mathrm{KL}}\left(\alpha_{k},1/n\right)<D_{\mathrm{KL}}\left(\alpha_{k},x_{2,k}\right)$. Intuitively, if $\left\{\alpha_{k}\right\}$ has a high increasing gradient, then we would choose $\left\{x_{2,k}\right\}$, the increasing distribution to represent it, but if $\left\{\alpha_{k}\right\}$ becomes smoother, then we would choose $\left\{x_{1,k}\right\}$, the uniform distribution.

As an example, suppose the two distributions {0.3,0.7},{0.4,0.6} have to be represented by one of the distributions {0.33,0.67},{0.5,0.5}. To preserve the order, we would choose {0.33,0.67} for both. It is intuitively a clear choice of {0.3,0.7} (and a correct one, as it has a K-L divergence, half the one for {0.5,0.5}), but we might have some doubts for {0.4,0.6}, where, actually, the K-L divergence is only slightly smaller for {0.5,0.5}. To represent distributions {0.43,0.57},{0.45,0.55}, it is intuitively more clear to select the uniform distribution {0.5,0.5} rather than {0.33,0.67}, even if order is lost. In fact, the K-L divergence from both distributions to {0.33,0.67} is one and two orders of magnitude greater, respectively, than to the uniform distribution {0.5,0.5}.

### 4.2. Relationship between K-L Dominance and First Stochastic Dominance Orders

Summarizing K-L dominance and first stochastic dominance orders, we have

1. $\left\{\alpha_{k}\right\}{≻}_{st}\left\{\alpha_{k}^{\prime }\right\}↔\forall x_{k}\;\mathrm{increasing}\;CE\left(\alpha_{k},x_{k}\right)\leq CE\left(\alpha_{k}^{\prime },x_{k}\right)$
2. $\left\{x_{k}\right\}{≻}_{\mathrm{KL}}\left\{x_{k}^{\prime }\right\}↔\forall \alpha_{k}\;\mathrm{increasing}\;CE\left(\alpha_{k},x_{k}\right)\leq CE\left(\alpha_{k},x_{k}^{\prime }\right)$

Is there a relationship between the two orders? When $\Pi_{k}x_{k}=\Pi_{k}x_{k}^{\prime }$ or, equivalently, $\sum_{k}\log x_{k}=\sum_{k}\log x_{k}^{\prime }$ (observe that Theorem 1 is a particular case), we can find a necessary and sufficient condition for $\left\{x_{k}\right\}{≻}_{KL}\left\{x_{k}^{\prime }\right\}$.

**Theorem** **6.** *Given distributions $\left\{x_{k}\right\}$, $\left\{x_{k}^{\prime }\right\}$, and $\Pi_{k}x_{k}=\Pi_{k}x_{k}^{\prime }$, then the following conditions are equivalent:*

*(1)*  (9) $$\left\{x_{k}\right\}{≻}_{\mathrm{KL}}\left\{x_{k}^{\prime }\right\}$$
*(2)*  (10) $$\left\{\frac{-\log x_{k}^{\prime }}{-\sum_{k}\log x_{k}^{\prime }}\right\}{≻}_{st}\left\{\frac{-\log x_{k}}{-\sum_{k}\log x_{k}}\right\}$$

**Proof.**  Observe first that $\left\{\frac{-\log x_{k}^{\prime }}{-\sum_{k}\log x_{k}^{\prime }}\right\},\left\{\frac{-\log x_{k}}{-\sum_{k}\log x_{k}}\right\}$ are also distributions (we can indeed use the simpler expressions $\left\{\frac{\log x_{k}^{\prime }}{\sum_{k}\log x_{k}^{\prime }}\right\},\left\{\frac{\log x_{k}}{\sum_{k}\log x_{k}}\right\}$, but, here, we have left the negated ones to remind the reader that both numerator and denominator are negative), because they are positive sequences adding to 1. Observe also that when $\Pi_{k}x_{k}=\Pi_{k}x_{k}^{\prime }$, taking the logarithms in Equation (4) and changing the sign (which changes the direction of inequalities), we obtain condition (3) in Theorem 2, which is necessary and sufficient for $\left\{\frac{-\log x_{k}^{\prime }}{-\sum_{k}\log x_{k}^{\prime }}\right\}{≻}_{st}\left\{\frac{-\log x_{k}}{-\sum_{k}\log x_{k}}\right\}$. □

**Example** **4.** *Consider the distributions {1/4,1/4,1/2},{1/4,1/2,1/4} and their normalized negated logarithms {2/5,2/5,1/5},{2/5,1/5,2/5}, respectively. We have that $\left\{1/4,1/4,1/2\right\}{≻}_{KL}\left\{1/4,1/2,1/4\right\}$ and $\left\{2/5,1/5,2/5\right\}{≻}_{st}\left\{2/5,2/5,1/5\right\}$.*

## 5. Conclusions and Future Work

In this paper, we present new inequalities for cross entropy and Kullback–Leibler divergence. As cross entropy is the negated logarithm of likelihood, the inequalities also hold for likelihood, changing the sense of the inequality. First, we applied to cross entropy the rearrangement inequality and recent stochastic order invariance results for Kolmogorov weighted means, as likelihood is a weighted geometric mean. Then, we introduced another partial order, K-L dominance, that applies directly to K-L divergences, and we give the relationship between both orders.

In data retrieval, exploration, analysis, and visualization, sorting some data objects into an ordered list or display is a common task performed by users. In the past, the benefit of having a sorted list has been typically articulated qualitatively. We plan to use the theorems presented in this paper to help establish an information-theoretic explanation about the cost–benefit of ordering in data retrieval, exploration, analysis, and visualization. We will also investigate the application to machine learning. We will study whether K-L-dominance order supports invariance properties, in the same way as first stochastic order supports the order invariance for weighted means, and we will look for sufficient conditions for K-L-dominance order, similar to the one in Theorem 3 for first stochastic order. Finally, we will investigate extensions of K-L dominance order: for instance, when $\left\{\alpha_{k}\right\}\equiv \left\{\frac{f(k)}{\sum_{k}f\left(k\right)}\right\}$, f(x) increasing and concave/convex, in the same way as the extensions of first stochastic order.
